# MRI in the evaluation of plantar plate disease: diagnostic value of the “stress test”

**DOI:** 10.1186/s10195-024-00814-x

**Published:** 2024-12-24

**Authors:** Luca Giuliani, Carlo Ottonello, Alessandra Giuliani, Lucia Bondì, Paolo Ronconi, Valerio Tempesta, Patrizia Pacini, Vito Cantisani

**Affiliations:** 1 Innovative Biomedical Technologies in Clinical Medicine, Sapienza Universitiy, Rome, Italy; 2 Fisiocard Center, Rome, Italy; 3 Department of Psychology, University of Bath, Bath, UK; 4 Tor Vergata University, Rome, Italy; 5 Campus Biomedico, Rome, Italy; 6 Ospedale San Sebastiano Martire, Frascati, Italy; 7 Sapienza Universitiy, Rome, Italy

**Keywords:** Forefoot MRI, Stress test, Plantar plate, Dorsal subluxation of the metatarsophalangeal joint

## Abstract

**Introduction:**

The plantar plate, also called the plantar ligament, is a fibrocartilaginous structure found in the metatarsophalangeal (MTP) and interphalangeal (IP) joints. Our study aimed to evaluate the role of magnetic resonance imaging (MRI) performed with the patient in the standard position or with joint hyperextension (the “stress test”, ST) in the study of plantar plate (PP) disease that involves metatarsophalangeal joints.

**Materials and methods:**

All patients underwent forefoot MRI (Atroscan C, Esaote, Genoa, Italy), operating at 0.2 T. All patients first underwent a standard MRI examination (coronal T1 and T2 weighted image (WI) with fat suppression and axial and sagittal T2 WI); the examination was completed by performing a stress test (hyperextension of toes). The ST is an easy task to perform and is not time-consuming (requiring only one additional sagittal fast spin echo (FSE) T2-weighted MRI sequence; repetition time/ echo time (TR/TE): 3200/90 ms) for patients and operators. A 45°-dorsiflexion ST was performed for approximately 2.30 min, the time required to complete the sequence. No further diagnostic investigations were necessary; no patients underwent arthrography or arthro-MRI. The examinations were performed in a double-blind mode by two operators with proven experience in musculoskeletal radiology; no cases of intra-operator discordance were found.

**Results:**

Twenty-five patients were recruited into our study over a 2-year period; 15 were positive for metatarsal pain and 10 were controls. Before treatment (surgery), all patients displaying symptoms underwent evaluation. As a result, the imaging features accurately represented the natural and actual conditions of the lesions. Among the symptomatic patients, 11 out of the 15 exhibited a PP tear or dysfunction in both the standard position and the ST. Additionally, two out of the 15 individuals displayed a tear in the ST alone, with no indication of it in the standard position. In contrast, two out of 15 patients showed no evidence of a PP tear in either the standard position or the ST. However, these two patients demonstrated dorsal subluxation during the ST, likely due to micro-instability resulting from PP failure. In the asymptomatic patients, nine out of the 10 individuals were found to be negative for PP dysfunction. Only one out of the 10 patients exhibited dorsal subluxation solely in the ST, indicative of plantar plate dysfunction, but no evidence of a tear in the PP. In the asymptomatic patients, standard MRI provided a specificity of 100% and a high negative predictive value (NPV) (90%), while the latter increased with the ST (specificity and NPV equal to 100%). In symptomatic patients, standard MRI gave a sensitivity of 75% when assessing a PP tear, which increased to 100% with the ST; the sensitivity of standard MRI the evaluation of MF subluxation was 60%, but it reached 100% with the ST.

**Conclusions:**

In our study, by introducing the ST, the sensitivity in both the diagnosis of a PP tear and the evaluation of MTP subluxation reached 100% (a surgical assessment was performed on all positive patients for confirmation). Ultrasound has the advantage of being a non-invasive method. However, comparing the results of our study with the data available in the literature, ultrasound has a lower sensitivity and a negative predictive value. Also, ultrasound does not allow for the assessment of possible bone marrow oedema or the degree of concomitant arthritis. If other studies in the literature confirm these results, it will be possible to consider incorporating the ST into diagnostic practice in the future.

## Introduction

The plantar plate (PP), also called the plantar ligament, is a fibrocartilaginous structure found in the metatarsophalangeal (MP) joint.

The PP has its proximal insertion in the metatarsal head and its distal insertion in the base of the proximal phalanx [1]. It blends with the transverse metatarsal ligament at the MP joint. The dominant tributary for the proximal PP’s perfusion arises from the metatarsal pedicle [2].

The PPs of the metatarsophalangeal joints and the transverse metatarsal ligaments form a continuous band of strong ligamentous tissue across the forefoot. In a transverse section, each PP is seen to be anchored to its metatarsal head by the collateral ligaments [3].

Moreover, it is important to note that the PP has attachments to other structures. The distal plantar fascial aponeurosis attaches to the proximal aspect of the PP, while the deep transverse intermetatarsal ligament connects the PPs of adjacent MP joints along the plantar aspect of the metatarsals. Additionally, a shallow central groove on the plantar aspect of the PP accommodates the flexor tendons [4–6].

The PP supports the weight of the body and restricts dorsiflexion. The primary role of the PP is to provide stability to MP joints II, III, and IV. This is achieved through the multiple attachments and composition of the PP, which consists of fibrocartilaginous tissue. The PP also acts as a cushioning mechanism, dampening compression forces experienced during weight-bearing activities at the MP joints. Furthermore, the PP contributes to the resistance against tensile forces generated during the windlass effect. This occurs through the proximal attachment of the plantar fascia and subsequent dorsiflexion of the MP joints [7–10].

As reported in a study by Park et al. [2], the length of the PP pedicle differed among 16 lower limb samples from freshly frozen human adult cadavers. In the study, it the average length and width of the plantar plate pedicle (PPD) in the metatarsophalangeal (MTP) joint was measured. The average length and width of the PPD among all MTP joints were found to be 2.01 mm (range 1.35–3.27 mm) and 2.08 mm (range 0.92–3.7 mm), respectively. To be precise, in the second metatarsal, the average length and width of the PPD were 2.08 mm (range 1.46–2.71 mm) and 2.27 mm (range 1.53–3.38 mm), respectively. In the third metatarsal, the average length and width were 2.05 mm (range 1.35–2.94 mm) and 2.16 mm (range 0.92–3.7 mm), respectively. Lastly, in the fourth metatarsal, the average length and width were 1.90 mm (range 1.42–3.27 mm) and 1.86 mm (range 1.21–2.59 mm), respectively.

Pathologies related to the PP, such as metatarsalgia, plantar swelling, hammertoe deformity, and subluxation of the lesser toes (particularly the second and third), can cause significant discomfort and functional impairments. Inadequate treatment of PP pathologies can lead to long-term disability, deformities, and dysfunction.

While a clinical examination is typically the first step in evaluating PP disorders, an accurate diagnosis requires further assessment through magnetic resonance imaging (MRI).

MRI allows for the evaluation of PP integrity and the assessment of potential MTP joint instability, such as dorsal subluxation of the proximal phalanx, even in the absence of apparent physical trauma [11–15]. MRI also has the advantage of allowing the easy detection of potential associated findings, such as stress fractures, neuromas, bone marrow oedema, ligament or tendon injuries, and joint effusions.

Compared to MRI, dynamic ultrasound is an affordable point-of-care test that can be easily conducted in an outpatient setting; also, an unstable PP can be easily identified by conducting a dynamic Lachman test with direct ultrasound visualization.

Both MRI and ultrasound are valid diagnostic tests for the diagnosis of a PP tear. MRI has the advantage that it can also identify associated bony findings and the dorsal subluxation of the proximal phalanx. At the same time, ultrasound is an examination that can be easily conducted and allows the MP joint to be easily stressed. The aim of using the MRI stress test (ST) is to eliminate the main limitation of MRI compared to ultrasound: stressing the joint simulates the normal forces to which the joints of the foot are subjected, thus allowing the identification of small injuries that would otherwise be missed.

The aim of this study was to examine the diagnostic accuracy of MRI completed with an ST compared to traditional MRI, with the goal of both improving diagnostic accuracy and minimizing the number of uncertain cases that require surgical verification.

## Material and methods

### Selection of patients

Patients were referred to our centre (Fisiocard Medical Centre, Rome, Italy) following specialist orthopaedic evaluation and standard radiographic examination of the foot.

This study’s case group consisted of patients who underwent an orthopaedic examination for metatarsal pain and were subsequently referred to our research due to a suspected PP tear.

In our study, radiographic examination of the feet was always performed before MRI, in comparison with the dorso-plantar, latero-lateral and the oblique projections, under load.

Twenty-five patients were recruited into our study over 2 years: 15 who experienced metatarsal pain and 10 who were asymptomatic. Of the 15 symptomatic patients, seven had prevalent symptomatology at the second metatarsophalangeal, four had it at the third metatarsophalangeal, two had prevalent symptomatology at the first metatarsophalangeal, and two patients had it at the fourth metatarsophalangeal.

The average age of the symptomatic patients was 58.7 years, and the average age of the asymptomatic patients was 59.5 years (Table 1). The median age was about 60.0 years for both symptomatic and asymptomatic patients. Patients older than 80 or younger than 35 were not included in the sample. None of the patients had ever undergone foot/ankle surgery or had contraindications for performing MRI examinations.

**Table 1:** Patient data

	Symptomatic patients	Asymptomatic patients
Number	15	10
Mean age (years)	58.7	59.5

### MRI protocol and stress test

All patients underwent forefoot MRI (Atroscan C, Esaote, Genoa, Italy), operating at 0.2 T.

This low-magnetic-field machine is ideal for patient comfort. It has a dedicated coil for studying the ankle/foot, allowing patient comfort and making it suitable for claustrophobic patients.

All patients first underwent a standard MRI examination (coronal T1 and T2 WI with fat suppression and axial and sagittal T2 WI) completed with an ST (hyperextension of toes).

The ST is an easy task to perform and is not time-consuming (requiring only one additional sagittal FSE T2-weighted MRI sequence; TR/TE: 3200/90 ms) for patients and operators.

**Fig. 1:**
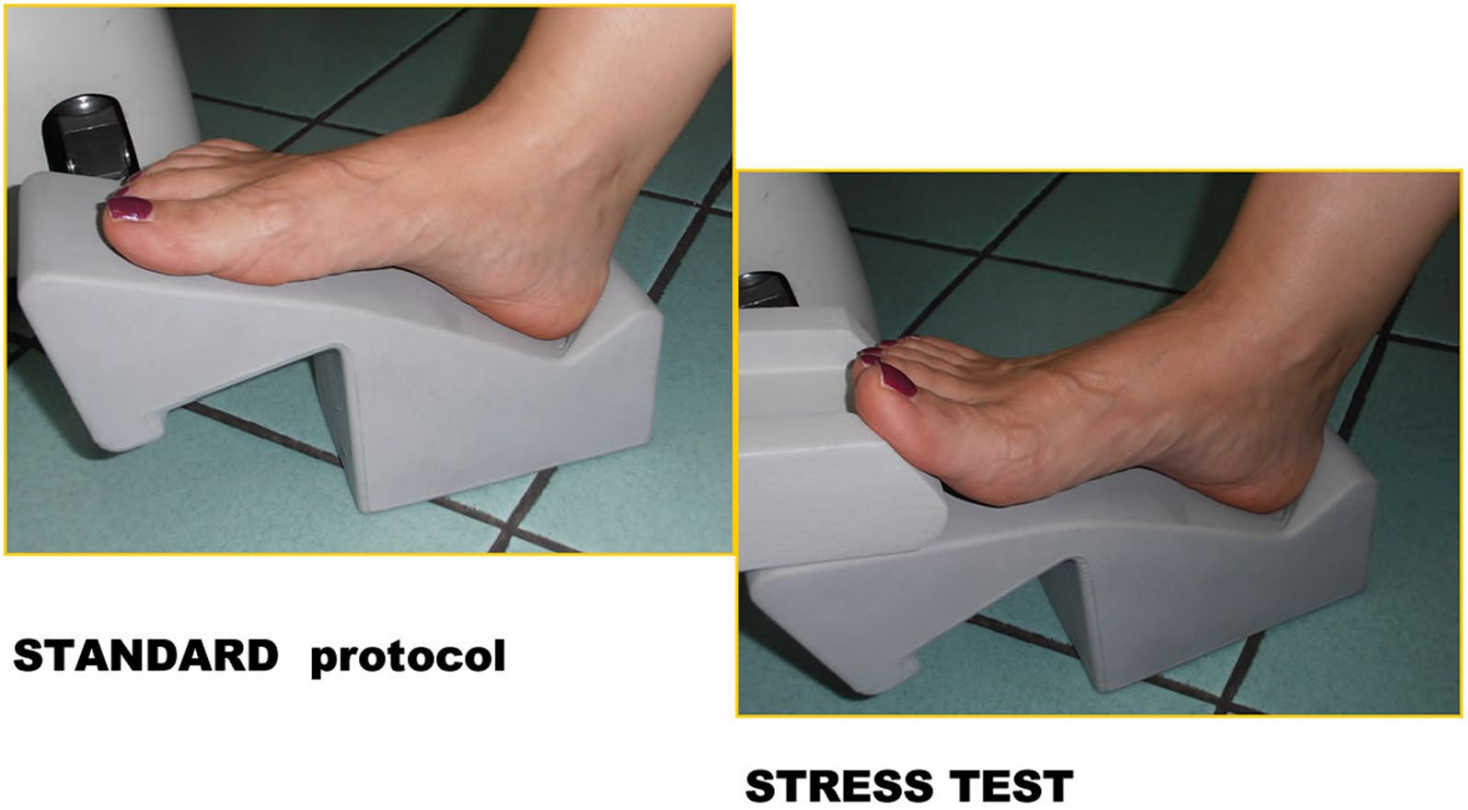
Standard position with joint hyperextension, and the stress test position

The standard execution of the examination, with the foot in a neutral position, is shown in Fig. 1 (on the left). In the ST, hyperextension of the toes is achieved by placing the PP itself in distension (Fig. 1, on the right).

The hyperextension condition was achieved by positioning a removable support. No device that would lead to forced hyperextension was employed (for example, a sling allowing hyperextension to the maximum degree was not used). Indeed, the use of such devices is a limitation, as they can reduce sensitivity in the case of a small PP tear. The support allowed for a good degree of hyperextension, ensuring patient stability without causing discomfort. Once the condition of maximum hyperextension was reached without causing discomfort, a reinforced pillow with a weight of approximately 2 kg was placed on the patient's leg to prevent any movement.

A 45°-dorsiflexion ST was then performed for approximately 2.30 min, the time required to complete the sagittal T2-weighted MRI sequence. The sequence obtained during the ST was subsequently analysed: a diagnosis of a PP tear was made if even a slight discontinuity of the normal hypointensity of the PP was observed; a diagnosis of MTP instability was made if a dorsal subluxation of the proximal phalanx in relation to the metatarsal head was observed.

No further diagnostic investigations were necessary; no patients underwent arthrography or arthro-MRI.

The examination also included an assessment of adjacent flexor tendons. Before treatment (surgery), all patients experiencing symptoms underwent evaluation. Consequently, the imaging characteristics accurately represented the lesions in their natural state.

The standard PP appears on MRI as homogeneously hypointense due to its fibrous main component. The flexor tendon runs just inferior to the PP, from which it is not always easily dissociable. This is why the MRI evaluation of a suspected PP tear must be performed by a radiologist with significant experience in the musculoskeletal field.

The examinations were performed in a double-blind mode by two operators with extensive experience in musculoskeletal radiology; no cases of intra-operator discordance were found. Fisher's exact test was used to identify statistically significant results.

## Results

We analyse the results of both symptomatic and asymptomatic patients separately.

Among *symptomatic patients*, 11 out of the 15 showed a PP tear in both the standard position and the ST.

Additionally, two out of the 15 patients exhibited a PP tear only in the ST, while it was not visible in the standard position.

On the other hand, two out of 15 patients did not have any PP tear in either the standard position or the ST. Despite the absence of a PP tear in these two patients, they exhibited dorsal subluxation of the proximal phalanx during the ST, indicating micro-instability associated with potential PP failure.

All 15 patients underwent surgical revision, which confirmed the MRI findings.

Among the *asymptomatic control group* (10 patients), 90% of the individuals yielded negative results for PP dysfunction in both the standard position and the ST. However, one participant (10%) showed positive indications of PP dysfunction as revealed by MRI. Notably, this individual exhibited dorsal subluxation exclusively during the ST. Nonetheless, no evidence of a PP tear was detected.

The surgical review confirmed the PP dysfunction highlighted in the MRI examination with the ST. The orthopaedic evaluation confirmed the negative results for the remaining nine patients and ruled out any PP dysfunction/tear.

No significant flexor tendon tear was detected in any patient (Table 2).

**Table 2:** Results

	Symptomatic patients	Asymptomatic controls
Tear in the standard position and the stress test	11/15	0/10
Tear only in the stress test	2/15	0/10
No plantar plate tear	0/15	9/10
No plantar plate tear with dorsal subluxation in the stress test	2/15	1/10

Some examples and images of the patients are provided below.

**Fig. 2:**
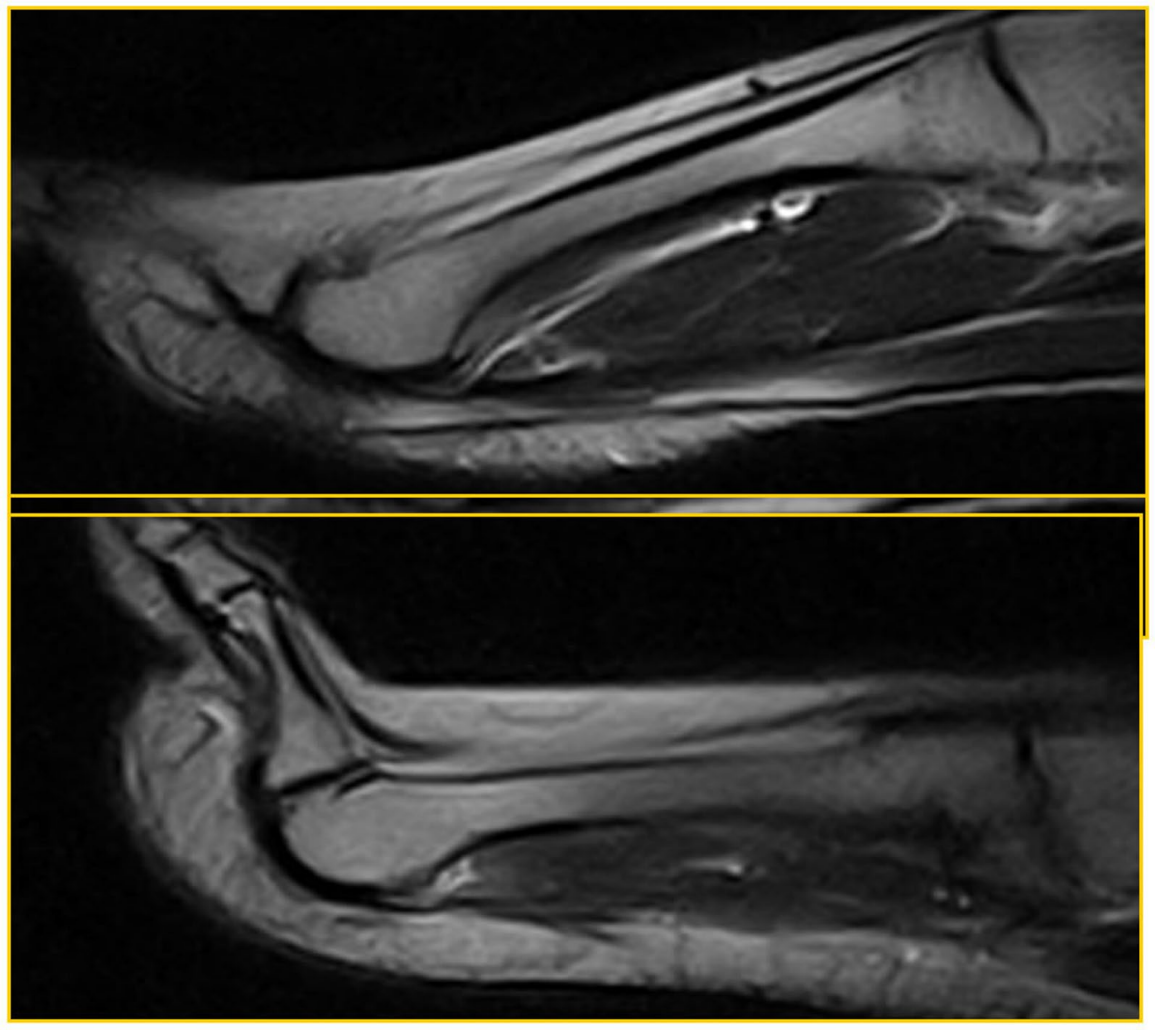
Patient 1: true negative

Patient 1 (a 52-year-old male) was asymptomatic and a true negative: he had a regular PP that was homogeneously hypointense in both the standard position and during the ST (Fig. 2).

**Fig. 3:**
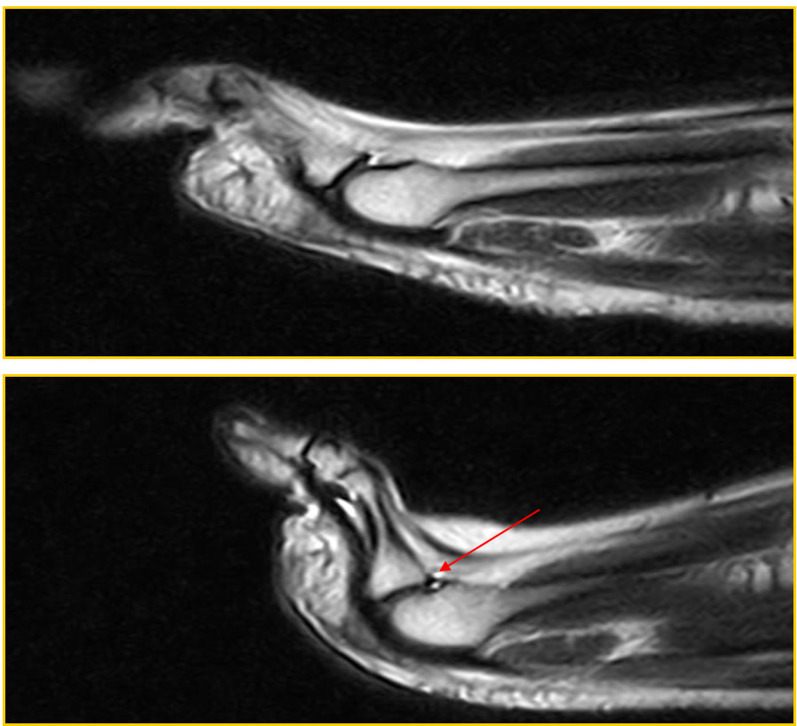
Patient 2: false negative (laxity)

Patient 2 (a 34-year-old woman) was asymptomatic and a false negative (laxity): she had MTP instability (dorsal subluxation of the proximal phalanx) that was only demonstrated during the ST in the absence of a PP tear (red arrow in Fig. 3).

**Fig. 4:**
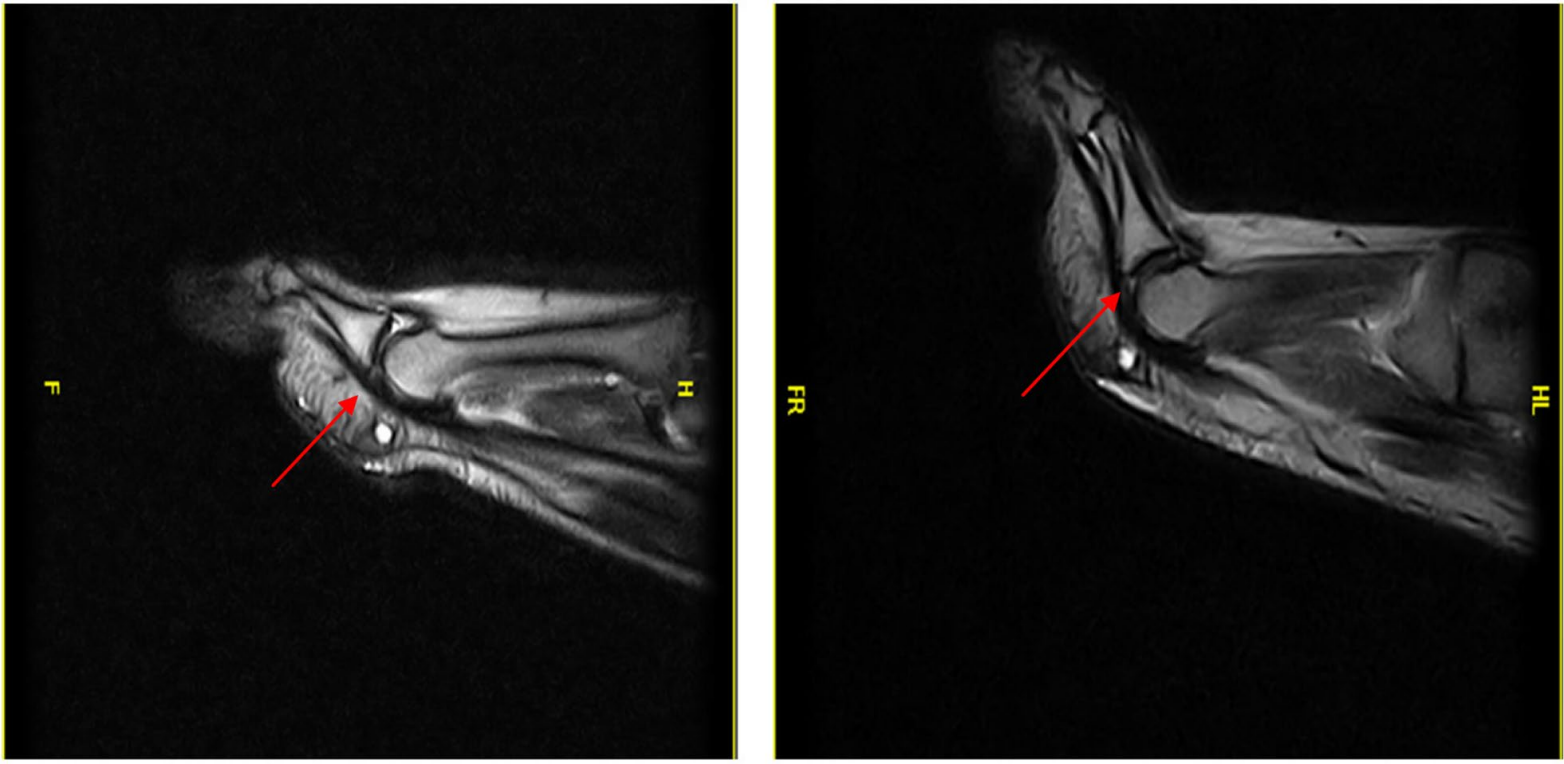
Patient 3: true positive

Patient 3 (a 55-year-old woman) was symptomatic and a true positive: a PP tear was visible both in the standard position and during the ST (red arrow in Fig. 4); dorsal subluxation was highlighted during the ST (Fig. 4).

In Fig. 4, the tear is seen to affect the distal portion of the PP at its insertion at the base of the proximal phalanx. There is a small superficial interruption of the plate's normal hypointensity.

**Fig. 5:**
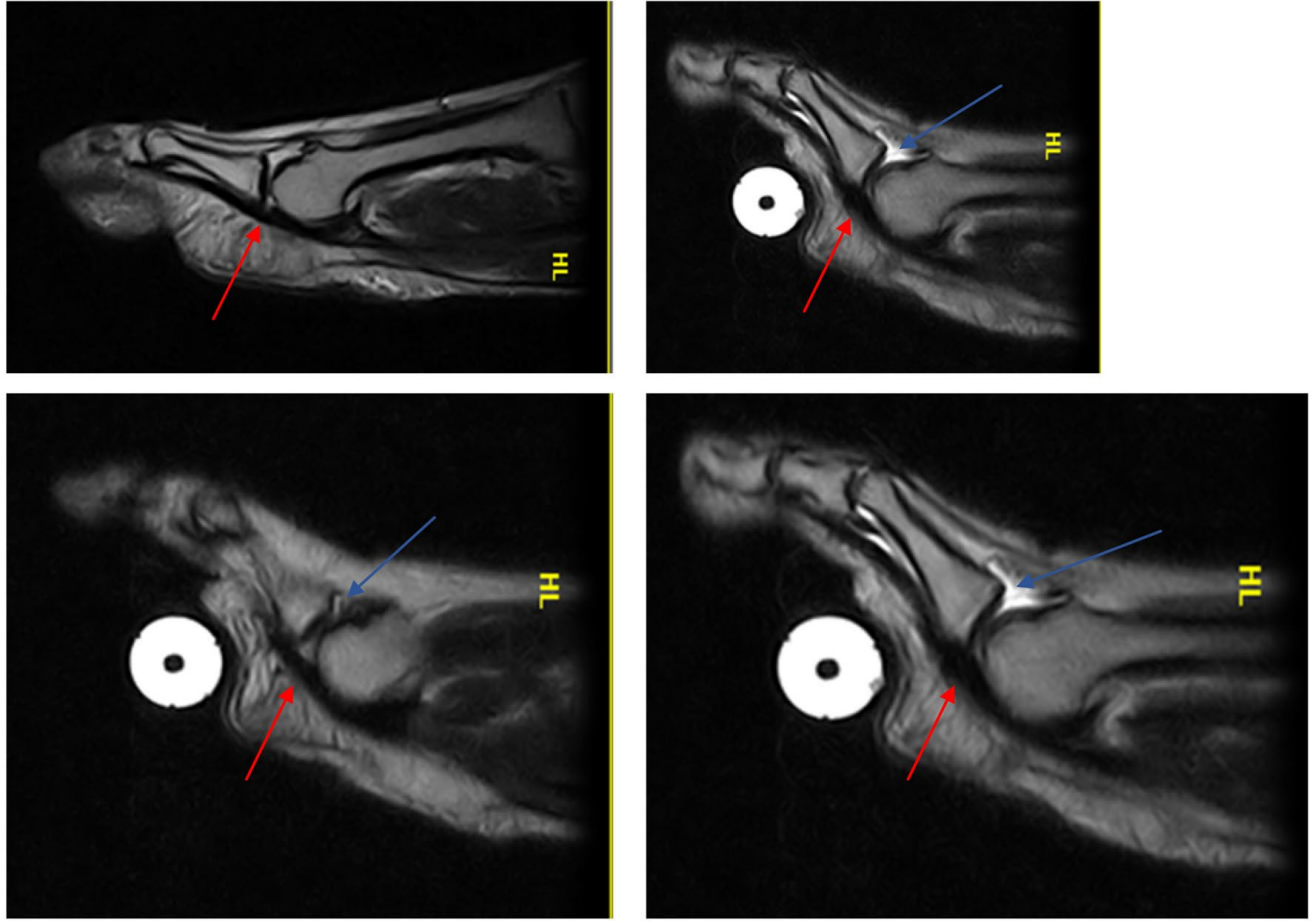
Patient 4: true positive

Patient 4 (a 53-year-old man) was symptomatic and a true positive: a PP tear was visible both in the standard position and during the ST (red arrow in Fig. 5); dorsal subluxation was highlighted in all slices during the ST (blue arrow in Fig. 5), with a fluid collection distending the dorsal capsular recess (Fig. 5).

The tear affected the distal portion of the PP at the insertion at the base of the proximal phalanx. 

**Fig. 6:**
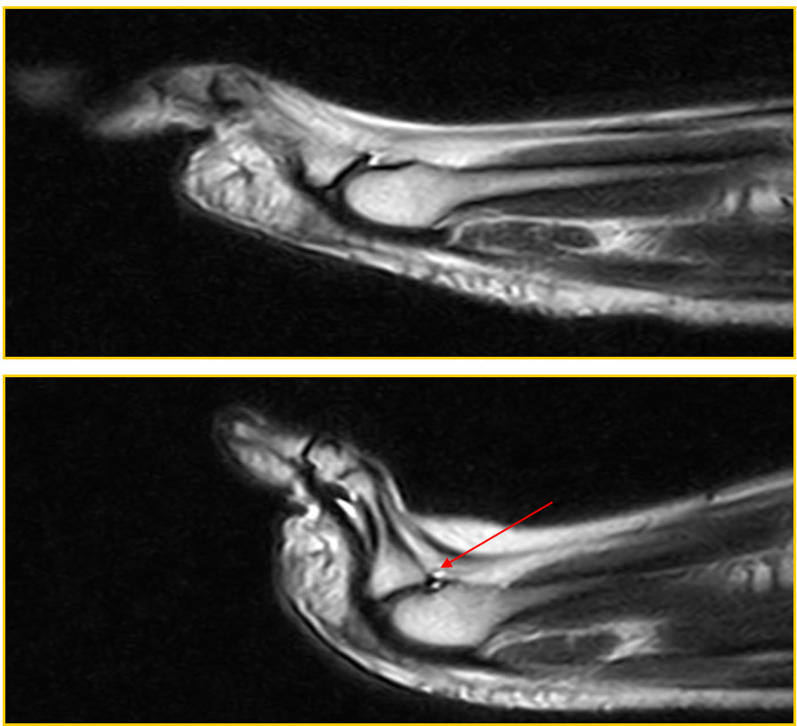
Patient 5: false negative (instability)

Patient 5 (a 59-year-old woman) was symptomatic and a false negative. She had joint instability: dorsal subluxation of the proximal phalanx, visible only during the ST (red arrow in Fig. 6), in the absence of a PP tear (Fig. 6).

**Fig. 7:**
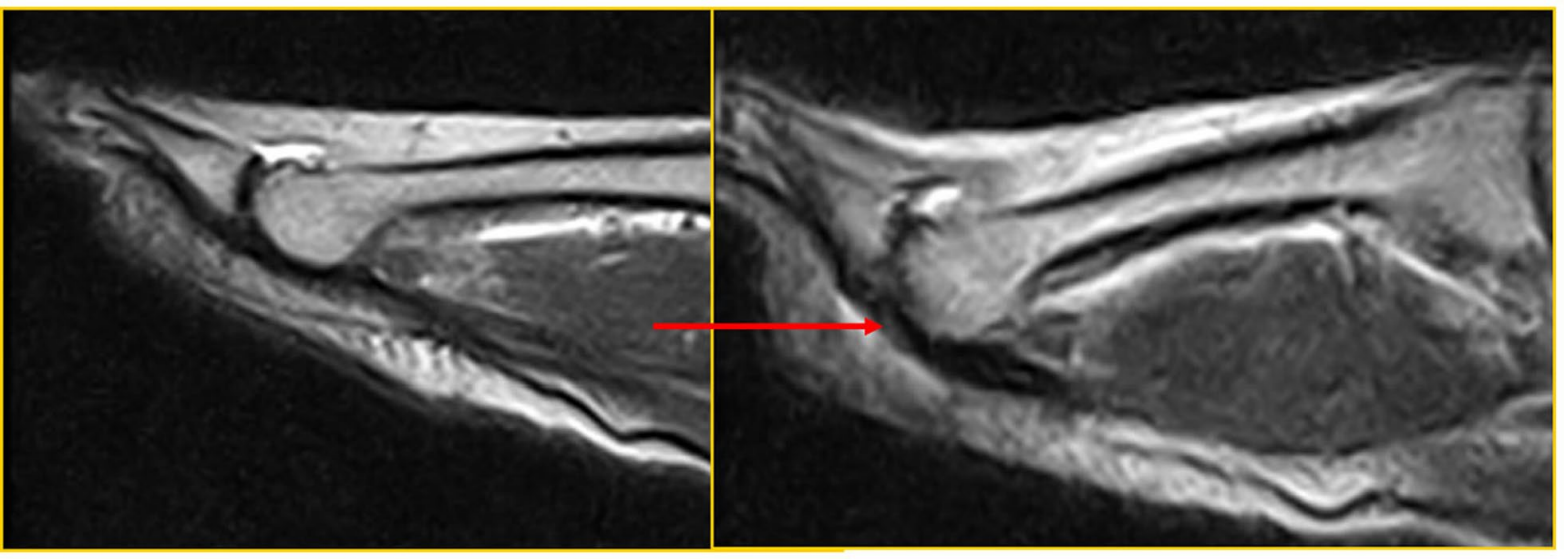
Patient 6: false negative

Patient 6 (a 54-year-old man) was symptomatic and a false negative: he was negative for a PP tear in the standard position. The tear became evident during the ST (red arrow in Fig. 7).

The PP tear, detectable only with the ST, affects the PP on the deep side at the insertion on the proximal phalanx.

**Fig. 8:**
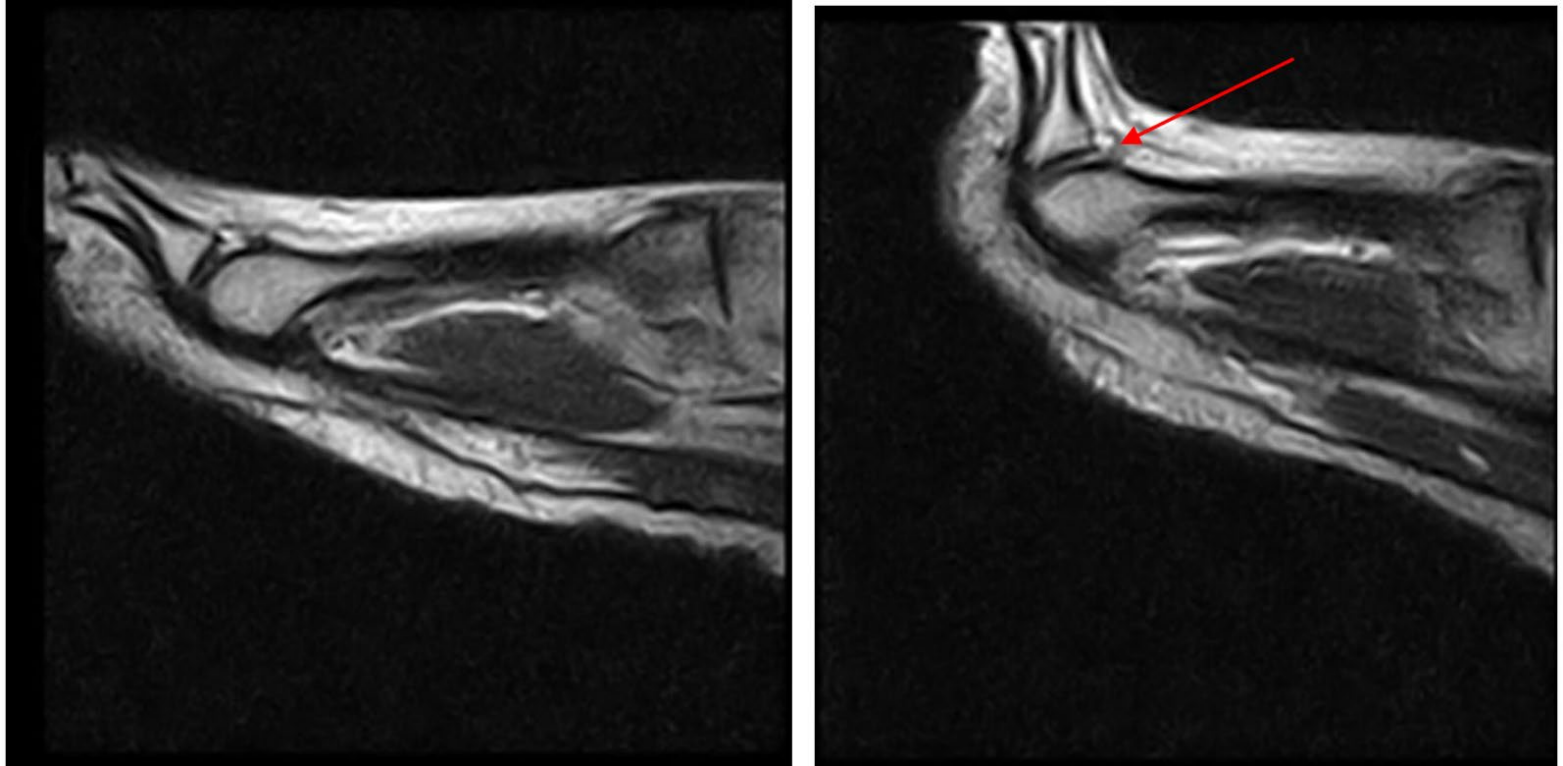
Patient 7: false negative (laxity)

Patient 7 (a 54-year-old man) was symptomatic and a false negative (laxity): he was negative for a PP tear in the standard position and during ST. Laxity in the absence of a tear (red arrow in Fig. 8) was later confirmed at revision surgery (Fig. 8).

The results show that stress testing is essential for correctly diagnosing PP tears in cases of a clinical suspicion of injury.

In asymptomatic patients, standard MRI had a specificity of 100% and a high negative predictive value (90%). The specificity and NPV increased to 100% with the ST.

In symptomatic patients, the assessment of a PP tear with standard MRI had a sensitivity of 75%, which increased to 100% with the ST. The sensitivity was 60% in the evaluation of MTP subluxation (standard MRI), but it reached 100% with the ST.

Therefore, it is important not only to identify misdiagnosed lesions but also to evaluate possible instability and dorsal subluxation of the proximal phalanx.

Among the 15 positive patients, an intermetatarsal space (IS) neuroma was observed in two cases (13.3%); second-IS-wide pericapsular fibrosis was observed in two cases (13.3%); and second and third MTP bursitis was observed in two cases (13.3%). Additionally, five positive patients (33.3%) showed bone lesions (one hadFreiberg disease, a form of avascular necrosis in the metatarsal bone of the foot, one had a stress fracture in the fifth metatarsal head, and three had first MTP degenerative arthritis). Finally, the remaining seven positive patients (46.6%) had a flexor tendinopathy. None of these alterations were found in asymptomatic patients.

Our study's advantage is that there was a surgical evaluation of all positive patients which confirmed our results.

## Discussion

In our study, ST MRI resulted in 100% sensitivity and specificity in identifying a PP tear in both symptomatic and asymptomatic patients. The standard MRI examination without a ST has a sensitivity rate of 75% for a PP tear and 60% for MTP subluxation evaluation in symptomatic patients.

Among the non-invasive methods used in the diagnosis of PP disease, ultrasound and standard MRI currently play equally important roles. Various studies and systematic reviews [11, 16–21] on the greater diagnostic validity of one or the other technique, often with non-univocal results, are available in the literature. However, both methods have limitations in identifying all PP tears.

A recent study by Donegan et al. [16] compared the diagnostic accuracy of MRI and high-resolution dynamic ultrasonography in evaluating PP pathology, with surgical confirmation as the reference standard. The study included a multicentre retrospective analysis. The study's findings indicated that MRI had a sensitivity of 60%, specificity of 100%, positive predictive value of 100%, and negative predictive value of 33%. The overall diagnostic accuracy of MRI, compared to intraoperative findings, was 66%. On the other hand, high-resolution dynamic ultrasonography showed a sensitivity of 100%, specificity of 100%, positive predictive value of 100%, and negative predictive value of 100%. Compared to intraoperative findings, the overall diagnostic accuracy of ultrasonography was 100%. Although it was not statistically significant, high-resolution dynamic ultrasonography demonstrated greater accuracy than MRI in diagnosing PP pathology in the lesser MP joint.

Contrary to the current study's findings, a meta-analysis conducted by Duan et al. revealed different results [17]. The meta-analysis included studies that investigated the diagnostic detection of MRI or ultrasound for PP tears, with surgical criteria used as the reference test. A total of seven studies, comprising 246 PP tears, were included in the meta-analysis. The results of the meta-analysis indicated that MRI exhibited higher diagnostic accuracy than ultrasound for the detection of PP tears. The sensitivity, specificity, positive likelihood, and negative likelihood ratios for MRI were 95%, 54%, 2.08, and 0.08, respectively. In comparison, ultrasound had a sensitivity, specificity, positive likelihood ratio, and negative likelihood ratio of 93%, 33%, 1.20, and 0.35, respectively. The summary receiver operating characteristic curve also indicated that MRI had superior diagnostic accuracy compared to ultrasound.

Albright et al. [18] conducted a systematic review and meta-analysis to assess the diagnostic accuracy of MRI and dynamic ultrasound for PP tears. The review included studies published in databases such as MEDLINE, CINAHL, and Clinicaltrials.gov until May 2020. MRI's pooled sensitivity and specificity for PP tears were 89% and 83%, respectively. Results showed that the sensitivity and specificity for ultrasound were 95% and 52%, respectively. Overall, MRI demonstrated superiority over ultrasound in diagnosing PP tears. However, ultrasound was more sensitive than MRI, suggesting that a negative ultrasound result would likely rule out a PP tear when there is uncertainty based on a physical examination alone.

In another comparative study, Klein et al. [11] examined the diagnostic performance of MRI and ultrasound in 51 patients with a suspected unilateral PP tear, specifically at the second metatarsophalangeal joint. All 51 patients were examined intraoperatively, and 46 PP tears were identified. The sensitivity, specificity, positive predictive value, and negative predictive value of MRI were 73.9%, 100%, 100%, and 29.4%, respectively. For ultrasound, the corresponding values were 91.5%, 25%, 91.5%, and 25%, respectively. Interestingly, MRI detected four collateral ligament tears that were not identified on ultrasound. Based on these results, both MRI and ultrasound are considered suitable modalities for imaging PP pathology.

A study conducted by Sung et al. [19] investigated the concordance between MRI findings and intraoperative results in a cohort of 41 patients (38 females and three males; 45 feet) with an average age of 52.1 years. Intraoperatively, 41 cases of PP tear and four cases of intact ligaments were identified. The assessment of accuracy, sensitivity, specificity, positive predictive value, and negative predictive value yielded values of 96%, 95%, 100%, 100%, and 67%, respectively. Moreover, the clinical diagnosis of PP injury achieved a high level of accuracy, with the condition correctly identified in 41 out of 45 patients (91%) within the study population.

Nery et al. [20], in their study, compared the evaluation of MTP plantar plates with arthroscopic findings in 35 patients. Using the standard examination, they observed a level of precision of 88.5% in senior radiologists and 77.0% in less experienced radiologists.

Yamadà et al. [21] investigated the diagnostic performance of direct and indirect MRI features for PP tears at II and III MTP, using surgical findings as the reference standard. The study encompassed a retrospective analysis of 23 patients who presented with symptomatic instability of lesser MTP joints and had previously undergone preoperative 1.5-T MRI and a subsequent surgical evaluation. A total of 45 lesser MTP joints were included in the analysis. The researchers found that the presence of pericapsular fibrosis exhibited high sensitivity (91.2%), specificity (90.9%), and accuracy (91.1%) for the diagnosis of PP tear. Furthermore, they determined that the PP–proximal phalanx distance yielded a sensitivity of 64.7%, specificity of 90.9%, and accuracy of 71.1% in diagnosing PP tears when a cutoff value of 0.275 cm was applied.

Even in MRI studies with higher levels of precision, an accuracy of 100% is never achieved. Our study aimed to evaluate whether a simple additional sequence could be a valid tool to achieve greater accuracy in diagnosing both PP tears and dysfunction.

Crucially, among non-invasive methods, forefoot MRI is not an operator-dependent investigation, and it provides more detail when compared with ultrasound, particularly on bone pathology. Compared to ultrasound, however, its current limitation is the inability to provide a dynamic evaluation simulating the normal load to which the MP joints are subjected.

The ST appeared to significantly increase the diagnostic accuracy of MRI in evaluating PP disease compared to the standard examination in our study and to previously reported data from other studies. In our opinion, the ST should not always be performed, but it should be performed in cases where there is a clinical suspicion of a lesion or instability of the PP or when a small PP tear is noted during the standard examination or there is a doubt about it.

The ST is simple and quick to perform (since it is a single sagittal T2-weighted sequence lasting about 2.30 min) for both the operator and the patient. The ST is a sequence that should be added at the end of the examination as a complement, without replacing the sagittal T2-weighted sequence in the standard examination. It has the advantage of negligibly increasing the overall duration of the exam, and it led to an improvement in both sensitivity and specificity in diagnosing a PP tear or dorsal subluxations of the proximal phalanx in our study. Potential limitations include possible poor patient compliance due to discomfort in maintaining the position or potential motion artifacts. It is important to note, however, that none of these situations occurred in any patient within our study sample.

Among the invasive diagnostic procedures, arthrography is now practically no longer required in the diagnosis of a PP tear, while the use of arthro-MRI is limited to doubtful cases due to the cost of the examination, despite the fact that distension of the joint with contrast media (MdC) allows the lesion to be easily identified.

When compared with arthro-MRI [22] (after the intra-articular injection of MdC, which is invasive), standard MRI + ST is a non-invasive and less expensive investigation that is also feasible with dedicated equipment.

Dinoà et al. [23] explored the difference between identifying a PP tear without and with contrast-enhanced and fat-suppressed MRI. This study examined a total of 249 contrast-enhanced forefoot MRI scans obtained from patients diagnosed with metatarsalgia between June 2012 and June 2013. The scans were reviewed by two specialized radiologists with expertise in musculoskeletal imaging. Among the patients included in the study, 59 individuals were identified as having a PP tear; it is noteworthy that 59% of these were female. Most patients with a PP tear (81.4%) had a single tear in one of their MTP joints. However, a small number of patients (seven out of 59) had a PP tear in each foot, three out of 59 patients had two PP tears in one foot, and only one out of the 59 patients had three lesions in one foot. Pericapsular fibrosis was observed in 70.5% of the PP tears in pre-contrast sequences. However, for the remaining 29.5% of the lesions, the presence of the tear was only evident after administering a contrast agent containing gadolinium. This highlights the substantial proportion of PP tears that became visible on MRI scans only after the administration of gadolinium.

In this case, the use of contrast agents increased the diagnostic capacity of the examination. Yet, the cost and potential danger of using contrast agents and the contraindications for an MRI examination make it an undesirable option.

Our study therefore shows the potential to reach a diagnosis of a PP tear with the simple addition of the ST when using any type of MRI machine. Ours is a preliminary study with a limited sample; although the results are very promising, other similar studies that can confirm our results are required. Should other studies report similar values of high sensitivity and specificity in the diagnosis of a PP tear with the use of the ST, there would be many advantages in normal diagnostic practice, such as being able to confirm or reject a clinical suspicion of a PP tear.

Firstly, it could mean that any type of MRI machine could be used, so there would not be the need for high-magnetic-field machines, which are often a prerequisite in the case of arthro-MRI or MRI with contrast agents. An MRI study with a sectoral system would also allow us to reduce the not-inconsiderable percentage of claustrophobic patients.

Furthermore, the duration of the examination would not increase significantly, making the examination easily reproducible.

MRI reveals information that cannot be obtained with ultrasound, allowing, first and foremost, the study of any concomitant bone pathology (e.g. bone marrow oedema). However, ultrasound can allow more reliable dynamic tests in real time. With the ST, which places a similar tension on the joint to that which can be achieved with ultrasound, previously unrecognized lesions can be identified.

Currently, there is no other article in the literature that evaluates the diagnostic accuracy of the ST, so further investigations are necessary. The main limitations of our study are the small number of patients and the recruitment of positive cases (referred by orthopaedic colleagues with a specific clinical question: to evaluate a PP tear). This situation is not easily reproducible in routine clinical practice, where it is often difficult to formulate a specific clinical question due to the frequent coexistence of multiple findings (such as a PP tear, arthritis, and tenosynovitis), which can complicate the primary diagnostic hypothesis.

In the case of a PP tear, significant improvements in symptoms are achieved after surgical treatment.

Baker et al. [24] performed a systematic review and meta-analysis of PP repairs; a total of 12 studies with 537 PP tears were included in the analysis. It revealed a significant reduction in Visual Analog Scale (VAS) pain scores postoperatively, with a pooled mean change of − 5.24 (95% CI − 6.09 to − 4.39). These findings were consistent across all studies that reported VAS pain. Notably, the dorsal approach showed promising outcomes, displaying an improvement in both VAS pain scores and American Orthopaedic Foot and Ankle Society (AOFAS) scores, with relatively narrow 95% confidence intervals. The summary estimates generated for pain and function at the 1-year follow-up in patients undergoing direct dorsal repair further support the predictability and effectiveness of this approach.

## Conclusion

Based on the data available in the literature, the standard MRI examination of the PP has a similar sensitivity for identifying PP tear to ultrasound, with a sensitivity rate varying between 60 and 90% in the various studies.

The aim of our study was to determine if MRI used with the ST has a significantly higher sensitivity and negative predictive value compared to MRI without the ST. This technique is a suitable noninvasive diagnostic method for identifying a PP tear or dysfunction (dorsal subluxation) with very low healthcare costs and total patient comfort (there is the possibility of using low-magnetic-field MRI equipment). A 45°-dorsiflexion ST was performed for approximately 2.30 min, the time required to complete the sequence.

In our study, introducing the ST increased the sensitivity for the diagnosis of a PP tear and the evaluation of MTP subluxation to 100%. Consequently, the results suggest that the ST may play a role in the detection of PP tears.

Ultrasound has the advantage of being a noninvasive method. However, upon comparing the results of our study with the literature, ultrasound appears to have lower sensitivity and a lower negative predictive value than MRI with the ST. Also, this methodology does not allow the possibility of bone marrow oedema or the degree of concomitant arthritis to be assessed. Furthermore, unlike MRI examinations, ultrasound examinations are operator dependent.

The major limitation of this study is the small number of patients, and further investigation is needed to confirm our preliminary data. In addition, great care must be taken with the device that allows hyperextension of the fingers to achieve a standardized degree of extension that is as reproducible as possible.

Ours was an initial study that showed the enormous potential of using the ST and its advantages. The sample size of our study was limited, so we emphasize the need for further studies to verify the possibility. Should further studies confirm our encouraging results, incorporating the ST into diagnostic practice in the future could be considered.

## Data Availability

The datasets used and/or analysed during the current study are available from the corresponding author on reasonable request.
